# Humoral immune response in adult Brazilian patients with Mucolipidosis III gamma

**DOI:** 10.1590/1678-4685-GMB-2018-0246

**Published:** 2019-11-14

**Authors:** Fernanda Sperb-Ludwig, Taciane Alegra, Renata Voltolini Velho, Nataniel Ludwig, Marina Siebert, Mariana Jobim, Filippo Vairo, Ida V. D. Schwartz

**Affiliations:** 1 Post-Graduation Program in Genetics and Molecular Biology, Universidade Federal do Rio Grande do Sul, Porto Alegre, RS, Brazil.; 2 BRAIN Laboratory, Experimental Research Center, Hospital de Clínicas de Porto Alegre, Porto Alegre, RS, Brazil.; 3 Medical Genetics Service, Hospital de Clínicas de Porto Alegre, Porto Alegre, RS, Brazil.; 4 Section Biochemistry, Children’s Hospital, University Medical Center Hamburg-Eppendorf, Hamburg, Germany.; 5 Immunology Service. Hospital de Clínicas de Porto Alegre, Porto Alegre, RS, Brazil.

**Keywords:** Mucolipidosis III gamma, inborn error of metabolism, humoral immune response, B-cell functions

## Abstract

Mucolipidosis II and III (ML II and III) alpha/beta and ML III gamma are lysosomal diseases caused by GlcNAc-1-phosphotransferase deficiency. Previous data indicate that MLII patients have functionally impaired immune system that contributes to predisposition to infections.We evaluated the immunological phenotype of three Brazilian patients with ML III gamma. Our data suggest that the residual activity of GlcNAc-1-phosphotransferase in patients with ML III gamma is enough to allow the targeting of the lysosomal enzymes required for B-cell functions maintenance.

Mucolipidosis II and III (ML II and III) alpha/beta and ML III gamma are rare lysosomal diseases caused by N-acetylglucosamine-1-phosphotransferase deficiency (GlcNAc-1-phosphotransferase, EC 2.7.8.17), enzyme responsible for the synthesis of the mannose 6-phosphate (M6P) tag added to lysosomal hydrolases. These lysosomal hydrolases depend on M6P residues to enter into the lysosomes via M6P specific receptors that mediate their transport (Braulke *et al.*, 2013).

ML II and III have variable presentations and a broad clinical spectrum, being ML II the more severe and ML III gamma the milder phenotype. They occur due to biallelic *GNPTAB (*ML II and III alpha/beta) or *GNPTG* (ML III gamma) pathogenic variants. ML II patients can present recurrent respiratory infections and low levels of IgA, IgG, and IgM. Impaired antigen presentation and B cell maturation *in vitro* were observed in animal models and in four patients. MLII patients presented impaired immunoglobulin levels and antibody responses to vaccination, whereas Knock-in mice have impaired antigen processing and presentation, defects in B cell maturation, and antibody production (Otomo *et al.*, 2015). Herein, we aim report on the immune phenotype of Brazilian patients with ML III gamma.

Two male siblings, age 42 and 45 years-old, and one female, age 19 years-old, with ML III gamma, previously described by our group (Velho *et al.*, 2014, 2016) were included in the study. They have had the routine childhood Brazilian immunization schedule as follows: Patient A: Bacille Calmette Guerin (BCG), diphtheria, tetanus, whooping cough, polio, measles, mumps and rubella; Patients B and C: BCG, diphtheria, tetanus, whooping cough, polio, smallpox, rubella. A blood sample was collected, and the total immunoglobulins levels, IgG antibodies against Rubella, Measles, Herpes (by immunoturbidimetry), IL-6 (by ELISA), and Complement CH50 (by immunoenzymatic assay) were analyzed. A flow cytometry analysis for cell count and B and T cells immunophenotyping, and IgG subclasses determination (by nephelometry) were also performed in the patients’ samples.

Results are presented in Table 1. Patients A and B have slightly increased IgG levels. High levels of IgG may occur in monoclonal gammopathies such as multiple myeloma, primary systemic amyloidosis, monoclonal gammopathy of uncertain significance, and related disorders (Dispenzieri *et al.*, 2001). None of those were diagnosed in our patients. In a previous cohort of ML II patients, the IgG, IgA, and IgM immunoglobulins levels were lower than reference due to intravenous injection of immunoglobulin to prevent common infection, except for two patients who showed normal IgG levels. (Otomo *et al.*, 2015).

**Table 1:** Clinical summary and immunological findings in patients with Mucolipidosis III gamma.

	Patient A	Patient B*	Patient C*
Age (years)	19	42	45
*GNPTG* genotype (cDNA)	c.[244_247dup];[328G>T]	c.[328G>T];[328G>T]	c.[328G>T];[328G>T]
*GNPTG* genotype (protein)	p.[Phe83*];[Glu110*]	p.[Glu110*];[Glu110*]	p.[Glu110*];[Glu110*]
IL-6	<2pg/mL	<2pg/mL	<2pg/mL
IgA (RV: 70-400 mg/dL)	214	306	175
IgE (RV: <100 UI/mL)	30.4	116.8	63.1
IgM (RV: 40-2030mg/dL)	84	168	127
IgG (RV: 700-1600mg/dL)	1690	1837	1550
IgG1 (RV: 240-1083 mg/dL)	NI	928	692
IgG2 (RV: 123-550 mg/dL)	NI	552	523
IgG3 (RV: 28-134 mg/dL)	NI	40	57
IgG4 (RV: 8-89mg/dL)	NI	317	278
Herpes IgG (RV: >30)	NI	>30	>30
Measles IgG (RV: >2U/L)	4.1	7.7	<1
Rubella IgG (RV: >10U/mL)	89.3	458.3	385.2
Leukocytes (RV: 3600-11000/uL)	7530	4660	6850
Lymphocytes (RV: 1000-4500/uL)	3610	1440	1452
CD3+/CD4+ T Cells (RV: 28%-57%)	NI	47,7	39,5
CD3+/CD8+ T cells (RV: 10%-39%)	NI	34	36,4
CD4+/CD8+ ratio (RV: 0.9–2.9)	NI	1.4	1.1
CD19+ B cells (RV: 3%-8%)	NI	3,9	4,2
CD20+ B cells (RV: 4%-23%)	NI	3,9	4,2
Complement CH50 (RV:>60U/CAE)	NI	133	138

Patients B and C presented increased levels of IgG4. Elevation in serum IgG4 concentration may be related to a wide variety of conditions, such as sarcoidosis which were not present in our patients, however, it may be elevated as a result of an undiagnosed allergy (Michel *et al.*, 2011; Wolfson *et al.*, 2017). Patient B had borderline levels of IgG2 and CD20+ B cells, and slightly elevated IgE which may be related to allergic causes as well. Primary immunodeficiencies, infections, malignancies, or other inflammatory diseases were not present in any of our patients.

Noteworthy, specific antibody response to vaccination was poor or not detectable in ML II patients and mice (Otomo *et al.*, 2015). However, it was not observed in our patients ML III. Patients B and C were not vaccinated against measleas, as it was not available in the national immunisation schedule until their adulthood. Although it is possible that patient B have had measles during his childhood. Also, the patients live in a small county with limited access to healthcare, which could be another cause for the lack of vaccination.

Herpes Simplex Virus (HSV) infections are common worldwide and the prevalence of HSV type 1 in Brazil is as high as 67.2% (Clemens and Farhat, 2010), so it was expected that HSV IgG antibodies would be found in immunocompetent individuals. All of our patients were positive for HSV IgG.

In all patients, IL-6 levels were below the detection limit. Interestingly, high levels of IL-6 has been reported in osteoblast and chondrocytes of ML II mice (Kollmann *et al.*, 2012).

This is the first study to evaluate immunological parameters in ML III patients. In our cohort, total leukocyte count and subpopulations were in the normal range, similar to what has been reported in four ML II patients (Otomo *et al.*, 2015). Besides that, ML III patients did not present recurrent infections, which can be explained both by normal immune system response and less severe affected airway compared to ML II patients, as airway obstruction caused by skeletal deformities, enlarged tongue, thickened mucosa, and larynx alterations are believed to contribute to predisposition to infections in ML II (Peters *et al.*, 1985).

B and T cells count were normal in our patients, however a decreased percentage of B cells and an increased percentage of T cells were observed by Otomo *et al.* (2015) in two patients with ML II. In summary, our data suggest that the residual activity of GlcNAc-1-phosphotransferase in patients with ML III gamma is sufficient to allow the targeting of the lysosomal enzymes required for B-cell functions maintenance, in contrast to the previously reports of patients and mice with ML II.
